# The role of stratospheric ozone for Arctic-midlatitude linkages

**DOI:** 10.1038/s41598-019-43823-1

**Published:** 2019-05-28

**Authors:** Erik Romanowsky, Dörthe Handorf, Ralf Jaiser, Ingo Wohltmann, Wolfgang Dorn, Jinro Ukita, Judah Cohen, Klaus Dethloff, Markus Rex

**Affiliations:** 1 Alfred Wegener Institute, Helmholtz Centre for Polar and Marine Research, Atmospheric Physics, Potsdam, 14473 Germany; 2 University of Potsdam, Institute of Physics and Astronomy, Potsdam, 14476 Germany; 3 Niigata University, Faculty of Science, Niigata, 950-2181 Japan; 4 Atmospheric and Environmental Research, Lexington, MA 02421 USA; 5 Massachusetts Institute of Technology, Department of Civil and Enviromental Engineering, Cambridge, MA 02139 USA

**Keywords:** Atmospheric chemistry, Atmospheric dynamics, Climate and Earth system modelling

## Abstract

Arctic warming was more pronounced than warming in midlatitudes in the last decades making this region a hotspot of climate change. Associated with this, a rapid decline of sea-ice extent and a decrease of its thickness has been observed. Sea-ice retreat allows for an increased transport of heat and momentum from the ocean up to the tropo- and stratosphere by enhanced upward propagation of planetary-scale atmospheric waves. In the upper atmosphere, these waves deposit the momentum transported, disturbing the stratospheric polar vortex, which can lead to a breakdown of this circulation with the potential to also significantly impact the troposphere in mid- to late-winter and early spring. Therefore, an accurate representation of stratospheric processes in climate models is necessary to improve the understanding of the impact of retreating sea ice on the atmospheric circulation. By modeling the atmospheric response to a prescribed decline in Arctic sea ice, we show that including interactive stratospheric ozone chemistry in atmospheric model calculations leads to an improvement in tropo-stratospheric interactions compared to simulations without interactive chemistry. This suggests that stratospheric ozone chemistry is important for the understanding of sea ice related impacts on atmospheric dynamics.

## Introduction

In recent decades, Arctic winter temperature has risen at more than double the rate of lower latitudes^1–3^ accompanied with a strong reduction of Arctic sea-ice extent^4^ and decreased sea-ice thickness^5^. This phenomenon called Arctic Amplification can lead to a weakening of the temperature gradient between the Arctic and the midlatitudes. A weakened temperature gradient is related to a meridionalisation of the atmospheric flow and increased advection of warm air into the Arctic^6^. In addition, dry cold polar air is transported into the midlatitudes, which can result in cold air outbreaks in Eurasia and North America. Advection of warm air leads to a reduction of Arctic sea ice and therefore an increased transport of heat and momentum into the atmosphere in fall and winter followed by an increase in wave propagation from the tropo- into the stratosphere and weakening of the stratospheric polar vortex^7,8^, which then affects the tropospheric circulation in the midlatitudes in subsequent months^9–11^. To understand this tropo- stratospheric interaction, an improvement of climate models physical mechanisms is essential^12,13^. Recent studies also have shown an impact of Arctic stratospheric ozone on the El-Nino Southern Oscillation through a link to the North Pacific Oscillation^14–16^. The purpose of this study is to evaluate the extent to which stratospheric ozone chemistry is important to climate linkages between the Arctic and midlatitudes. Therefor we implement a computationally fast but accurate interactive stratospheric ozone chemistry module into an atmospheric general circulation model, which allows to perform a large number of ensemble simulations.

## Model and Experimental Setup

Our study employs the atmospheric general circulation model (AGCM) ECHAM6^17^ version 6.3 with a spectral horizontal resolution of T63 (~1.875° longitude by 1.875° latitude on a Gaussian grid) and 95 vertical levels up to 0.01 hPa (~80 km). To isolate the impact of Arctic sea-ice retreat we perform sensitivity experiments only varying the sea-ice concentration (SIC). To simulate high-ice conditions (HICE) we use the average 1979 to 1983 and for low-ice conditions (LICE) the average 2005 to 2009 SICs from the Merged Hadley-National Oceanic and Atmospheric Administration/Optimum Interpolation sea surface temperature (SST) and SIC data set^18^. For all experiments we use the SSTs of the HICE period. We also reduce the sea-ice thickness (SIT) from 2 m in the HICE experiments to 0.5 m in the LICE setup, since changes in SIT in the Arctic can also contribute to atmospheric circulation changes^19,20^.

To investigate the interactions between ozone, sea ice and the atmospheric circulation we couple ECHAM6 with the fast but accurate interactive ozone chemistry scheme SWIFT^21^ and repeat the model experiments with HICE and LICE conditions. Each of these four perpetual model simulations is integrated over 120 years, excluding the first 20 years as a spin-up period from the analysis. Compared to ECHAM6 the newly coupled ECHAM6-SWIFT does not apply ozone climatologies in the stratosphere but solves a set of coupled differential equations, which simulate the polar vortex-averaged mixing ratios of the chemical key species involved in polar ozone depletion on each discrete model level that is located in the range between 20 hPa and 80 hPa. The stratospheric polar vortex edge is assumed to be located at the 36PVU contour line of the modified potential vorticity field^22^ at the respective model level. Compared to classic chemistry climate models this method can be applied to obtain very large sample sizes while being computationally feasible.

## Results

### The stratospheric pathway

As already discussed in recent literature, the ERA-Interim reanalysis dataset^23^ allows an attribution of changes of atmospheric dynamics to changed sea-ice conditions through the stratospheric pathway, when combined with the interpretation of model results^7–9,24^. To enable comparisons between the ECHAM6 model and reanalysis data, we select two time periods of the ERA-Interim data set representing high (winters 1979/80 to 1999/00) and low (winters 2000/01 to 2015/16) Arctic sea-ice conditions^8^.

**Figure 1:**
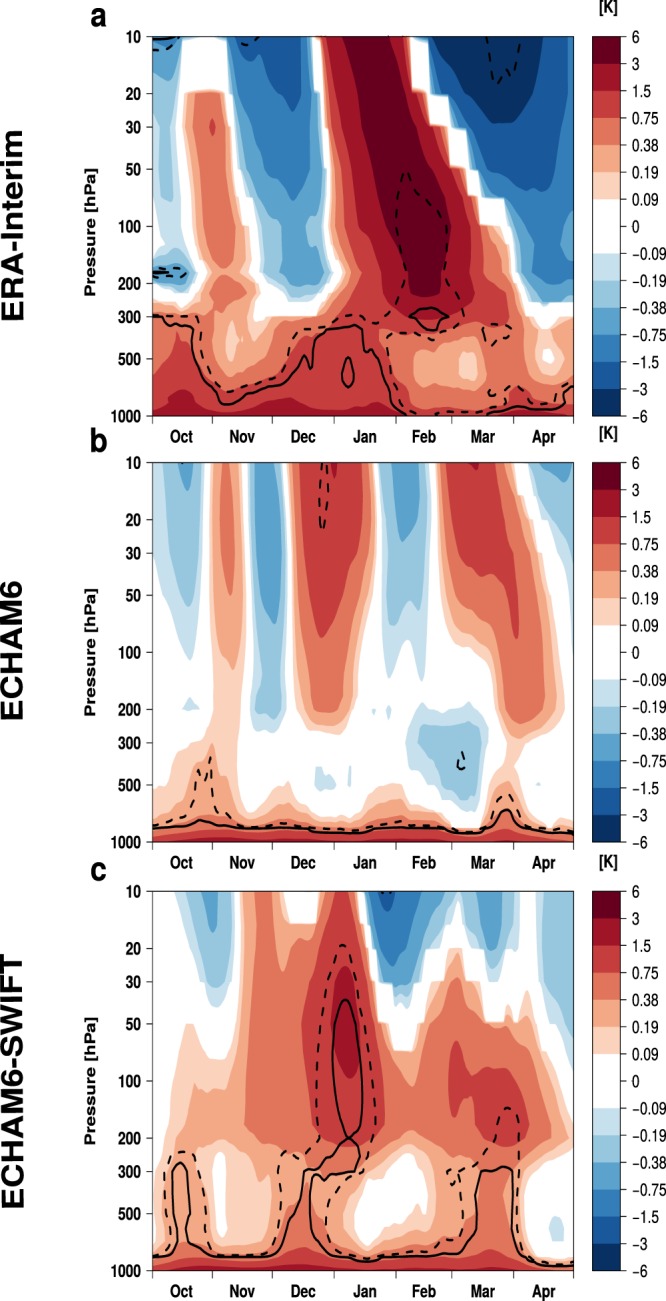
Time-height cross sections of climatological mean temperature differences [K] from 65°N to 90°N (LICE minus HICE) for ERA-Interim reanalysis data (**a**), ECHAM6 model simulations (**b**) and ECHAM6-SWIFT model simulations (**c**). Dashed/solid lines indicate statistical significance at the 95/99% level according to a two-sided students t-test.

In addition to a near surface warming that is also caused by the general Arctic warming due to climate change, ERA-Interim, starting in early January, shows a statistically significant positive polar cap (65° to 90°N, Fig. 1a) temperature anomaly propagating downward from the stratosphere into the upper troposphere for LICE minus HICE conditions, according to a standard two-sample two-sided students t-test. This indicates a weakening of the stratospheric polar vortex, which then can influence weather systems in the troposphere^25,26^. In the ECHAM6 model without the chemistry module, besides surface warming, a statistically significant response of the atmosphere due to sea-ice reduction is absent in the difference between the LICE and HICE experiments (Fig. 1b). ECHAM6-SWIFT, which takes into account interactive stratospheric ozone chemistry compares favorably to ECHAM6 with a more consistent stratospheric signal (Fig. 1c). Though the simulated downward propagation leads the observed downward propagation by two weeks.

### Upward wave propagation and ozone-dynamics interaction

**Figure 2:**
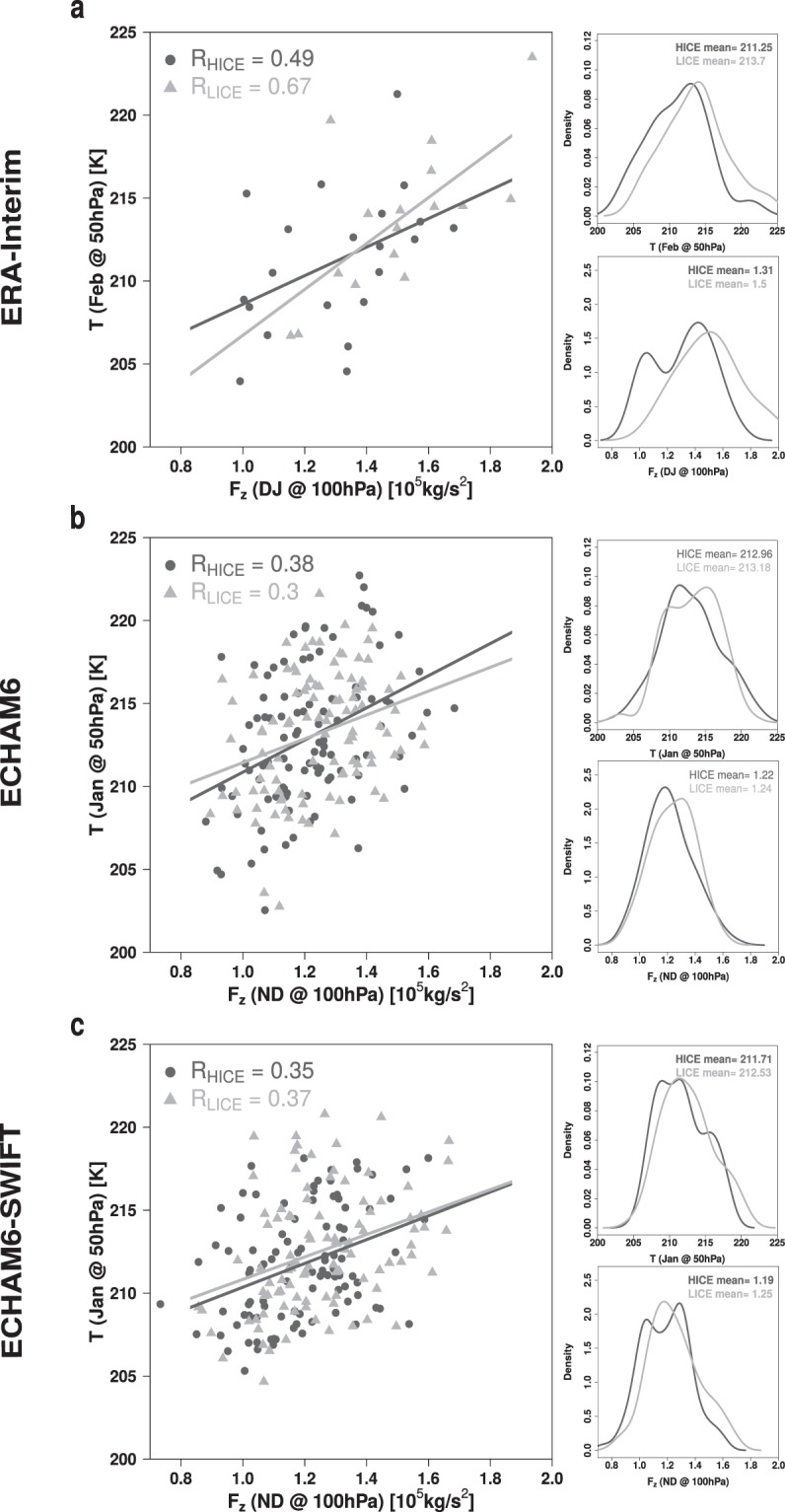
Relation between the vertical component of the EP-Flux [10^5^ kg s^−2^] in winter at 100 hPa between 45°N and 75°N and the polar cap (60°N to 90°N) mean temperature [K] at 50 hPa in the following months with PDFs for ERA-Interim reanalysis data (**a**), ECHAM6 model simulations (**b**) and ECHAM6-SWIFT model simulations (**c**). Bold mean values for the distributions means indicate a statistical significant difference between the LICE and HICE dataset according to a two-sided students t-test.

To understand how the stratospheric temperature signals are generated, we investigate the mean vertical transport of momentum by atmospheric waves through the tropopause region (100 hPa) between 45°N and 75°N before the maximum monthly mean polar cap temperature signal in 50 hPa occurs. The vertical component of the Eliassen-Palm (EP) flux vector (*F*_*z*_) in this region is an indicator for the momentum transported from the troposphere into the stratosphere by planetary waves^27,28^. Here, *F*_*z*_ is integrated over the two months preceeding the month showing the maximum temperature signal^29^ of each dataset, respectively. Its correlation with the stratospheric temperature is shown in Fig. 2. In the ERA-Interim reanalysis (Fig. 2a), the ECHAM6 model data (Fig. 2b) and the ECHAM6-SWIFT model data (Fig. 2c) clear relations of these two quantities are apparent. In all datasets higher values of *F*_*z*_ are related to higher stratospheric temperatures in the following months. All correlations are statistically significant at the 95% confidence level. Also in ERA-Interim the mean of the distributions of *F*_*z*_ at 100 hPa and temperature at 50 hPa are significantly different at the 95% confidence level when comparing the LICE and HICE conditions. In LICE *F*_*z*_ tends to be higher, indicating an enhanced propagation of planetary waves into the stratosphere. By the momentum deposited this way, the stratospheric circulation is disturbed, resulting in higher temperatures. While the LICE and HICE experiments for ECHAM6 show no statistically significant difference in the mean of these distributions, in the ECHAM6-SWIFT LICE and HICE experiments both, *F*_*z*_ and temperature, are significantly different. In contrast to ECHAM6, ECHAM6-SWIFT is able to simulate a qualitatively improved impact of Arctic sea-ice loss on the stratospheric winter circulation. This suggests that wintertime stratospheric dynamics and its impact on tropospheric wave behaviour^30^ can be improved by the implementation of interactive stratospheric ozone chemistry.

**Figure 3:**
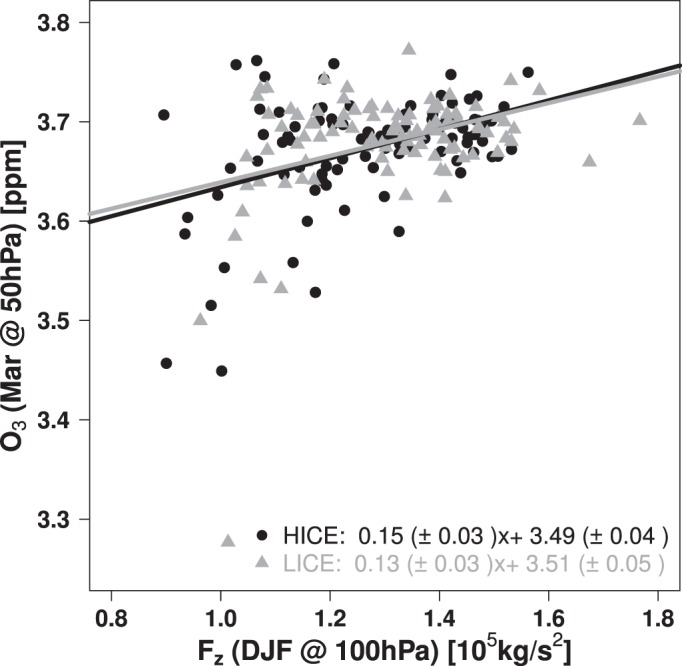
Relation between the vertical component of the EP-Flux [10^5^ kg s^−2^] in winter at 100 hPa between 45°N and 75°N and the polar cap (60°N to 90°N) mean ozone volume mixing ratio [ppm] at 50 hPa in the following March for ECHAM6-SWIFT.

One reason for the improved response is the interaction of propagating planetary waves with the stratospheric polar vortex and its dynamical relation to stratospheric ozone. In Fig. 3 the vertical component of the EP flux (*F*_*z*_) at 100 hPa between 45°N and 75°N and its connection to polar-cap stratospheric ozone in the following spring is shown. Momentum is transported through the tropopause to high latitudes where it is deposited. This disturbs the stratospheric polar vortex, leads to warming of the polar cap and to poleward and downward transport of ozone with the amplified residual circulation, resulting in the positive correlation between *F*_*z*_ and ozone^31^ in Fig. 3. Linear regression analysis for the ECHAM6-SWIFT HICE and LICE simulations shows that both relations are statistically significant exceeding the 95% confidence level, proving that wave forcing entering the stratosphere in winter impacts the stratospheric ozone volume mixing ratio (VMR) in spring, which impacts stratospheric temperature because of its radiative properties. Lower values of *F*_*z*_ result in lower polar cap temperatures which allows the formation of more Polar Stratospheric Clouds (PSC) leading to activation of chlorine species through heterogeneous reactions. With the return of sunlight this results in chemical destruction of ozone, further steepening the relation between polar cap ozone and *F*_*z*_, particularly for low values of *F*_*z*_ on the left hand side of Fig. 3, where the colder conditions allow for more significant chemical ozone losses. Because these mechanisms are ignored when ozone is prescribed, including the interactive ozone scheme SWIFT leads to clear improvements in the dynamical response of the model.

### Impact on tropospheric circulation patterns

**Figure 4:**
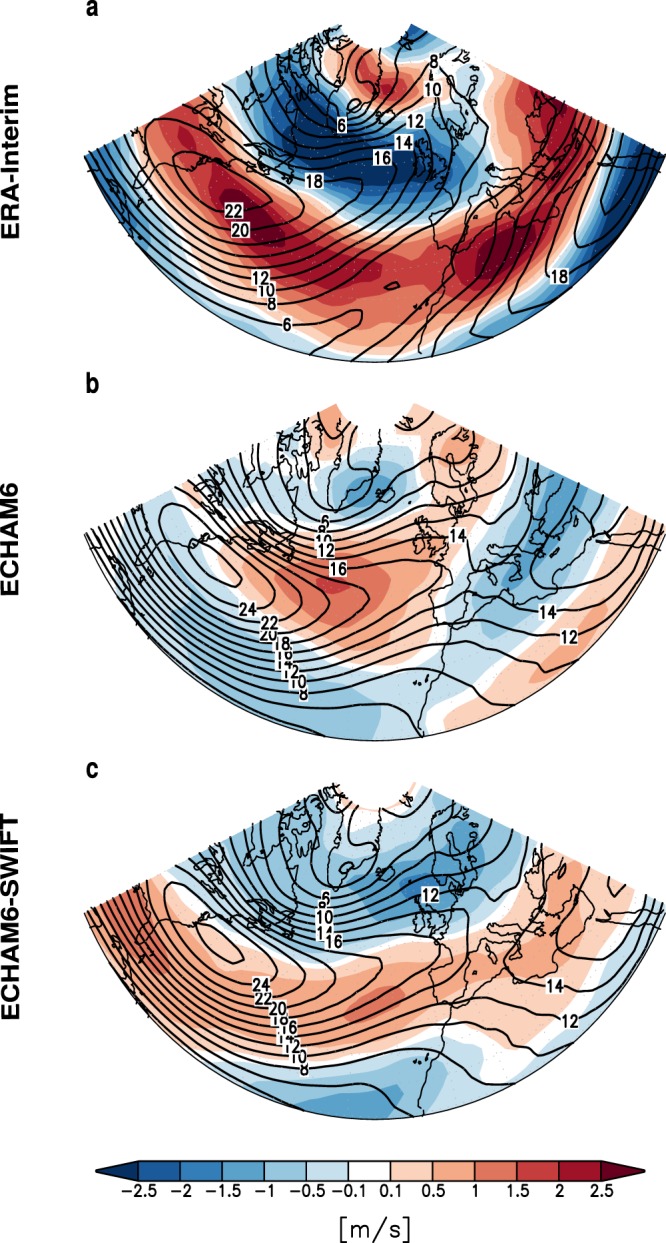
Climatological differences (LICE minus HICE) for the region of the NAO-Analysis in late winter (FM) zonal wind [m s^−1^] at 500 hPa and zonal wind climatologies of HICE (black contour lines) for ERA-Interim reanalysis data (**a**), ECHAM6 model simulations (**b**) and ECHAM6-SWIFT model simulations (**c**).

Several studies have shown that a reduction of Arctic sea ice leads to a negative phase shift of the North Atlantic Oscillation (NAO) in late winter^32–34^. In ECHAM6-SWIFT Arctic sea-ice reduction leads to a reduction of the zonal wind component in February/March (FM) at 10 hPa in the polar cap region of up to 3 m/s, which is of the magnitude of the induced forcing needed to significantly change the interaction between planetary waves and the mean flow^9^, whereas such a signal is absent in the ECHAM6 experiments without interactive stratospheric ozone chemistry. This change in the stratospheric circulation also leads to tropospheric anomalies in the following months, due to alterations in upward wave propagation. This can be seen in Fig. 4 where LICE minus HICE anomalies of the mean zonal wind in February-March at 500 hPa are shown for ERA-Interim reanalysis data (a), the ECHAM6 (b) and the ECHAM6-SWIFT (c) experiments. A weakening of the midlatitude westerly winds over the North Atlantic is evident in the reanalysis data. The wind change pattern in ECHAM6-SWIFT closely resembles the reanalysis, but is clearly different in ECHAM6.

To quantify changes in the tropospheric NAO we perform an Empirical Orthogonal Function (EOF) analysis for the fields of 500 hPa Geopotential Height from 90°W to 40°E and 20°N to 80°N^35^ for the period of the wind anomalies shown in Fig. 4. This analysis is performed by calculating the EOF and the principal components (PC)^36^ of the combined HICE and LICE datasets for the reanalysis data and each of the two model configurations. The corresponding NAO index for each dataset is defined as the leading EOFs mean PC over the respective dataset (e.g. ECHAM6-SWIFT HICE). Changes in NAO indices between LICE and HICE are represented by differences of this mean PCs. For ERA-Interim a strong negative shift in the phase of the NAO is found (PC difference LICE minus HICE: −0.42). Consistent with the zonal wind anomalies ECHAM6 shows no clear changes in the phase of the NAO, even tending to a positive shift (+0.07), while ECHAM6-SWIFT shows a clear tendency towards a negative NAO (−0.29). This shows that changes in Arctic sea ice can lead to a shifted circulation which increases the potential for cold air outbreaks over Europe, in agreement with the reanalysis.

## Conclusion

During LICE conditions ERA-Interim reanalysis data shows enhanced upward wave propagation from the troposphere into the stratosphere that causes a disturbance of the stratospheric polar vortex, which leads to downward propagating signals in the dynamical atmospheric variables. This process influences tropospheric circulation patterns like the NAO, impacting the daily weather patterns of the midlatitudes. This mechanism can not be fully reproduced by the AGCM ECHAM6, which does not respond with a negative phase shift of the NAO during LICE conditions. Coupling ECHAM6 to the fast but accurate interactive ozone chemistry scheme SWIFT improves the interaction between stratospheric chemistry and dynamics due to a more realistic representation of stratospheric processes. These improvements in the stratosphere also lead to a change in tropospheric teleconnection patterns resulting in a negative NAO response that is comparable to changes seen in reanalysis data.

Our results imply that interactive stratospheric ozone chemistry in winter and spring is important to understand changes in tropospheric teleconnection patterns caused by decreasing Arctic sea ice via the stratospheric pathway. Being computationally feasible, we also suggest the implementation of the fast ozone chemistry scheme SWIFT into other AGCMs enabling very large ensemble sizes compared to classic Chemistry Climate Models.

## Data Availability

Model data are available from the corresponding author on reasonable request.
